# Hypofractionated Stereotactic Radiotherapy for Patients with Intracranial Meningiomas: impact of radiotherapy regimen on local control

**DOI:** 10.1038/s41598-018-32124-8

**Published:** 2018-09-12

**Authors:** F. Meniai-Merzouki, V. Bernier-Chastagner, J. Geffrelot, E. Tresch, T. Lacornerie, B. Coche-Dequeant, E. Lartigau, D. Pasquier

**Affiliations:** 1 Academic Department of Radiation Oncology, Centre Oscar Lambret, 3 rue Combemale, 59020 Lille cedex, France; 2 Departement de radiotherapie, Institut de cancérologie de Lorraine, 6 Avenue de Bourgogne, 54519 Vandœuvre-les-Nancy, France; 3 Departement de radiotherapie, Centre François Baclesse, 3 Avenue du Général Harris, 14000 Caen, France; 4 Departement de biostatistique, Centre Oscar Lambret, 3 rue Combemale, 59020 Lille cedex, France; 5 Departement de physique médicale, Centre Oscar Lambret, 3 rue Combemale, 59020 Lille cedex, France; 6 CRISTAL, UMR CNRS 9189, Lille University 1, M3, Avenue Carl Gauss, 59650 Villeneuve-d’Ascq, France

## Abstract

We evaluated efficacy and tolerance of hypofractionated stereotactic radiation treatment (hFSRT) in the management of intracranial meningiomas. Between December 2008 and June 2016, 126 patients with 136 intracranial meningiomas were treated with robotic hFSRT. hFSRT was performed as primary irradiation and as a salvage option for the local recurrence after prior radiotherapy. The median prescription dose was 25 Gy (12–40) with a median number of fractions of 5 (3–10). After a median follow-up of 20.3 months (range 1–77 months), the 24-months local control (LC) rate was 81% in the primary hFSRT group and 39% after hFSRT in the re-irradiation group (p=0.002). The clinical control rate of symptoms in the overall population was 95% (95% CI: 89–98%). Progression-free survival (PFS) in the overall population at 24 months was 70% (95% CI: 60%–79%). In the primary hFSRT group, PFS was significantly lower with the most hypofractionated schedules of 21–23 Gy in 3 fractions vs. 25–40 Gy in 5–10 fractions: 62% vs. 92% (p = 0.0006). The incidence of radionecrosis at 24 months was significantly lower in the primary hFSRT group, at 2% vs. 20% in the re-irradiation hFSRT group (p = 0.002).

## Introduction

Meningiomas are the most frequent non-glial primary intracranial tumors in adults. They represent about 15–20% of all brain tumors^1^. The incidence of intracranial meningiomas, depending on the age of the patient, is estimated to be 8.4/100,000 in the elderly and 0.3 cases per 100,000 in childhood^2,3^. Most are usually benign in nature, with slow growth; studies estimate that annual growth averages 1–3 mm per year^4,5^. However, this characteristic does not make them harmless tumors, despite advances in diagnostic and therapeutic means. Indeed, meningiomas become aggressive by their topographies and evolutionary aspects. The main treatment strategy is maximal surgical resection, which should be as complete as possible while preserving the patient’s neurological status. Moreover, many meningiomas cannot be completely resected, or are unresectable because of their location adjacent to critical structures, making them difficult to remove safely^6^. Radiation can be delivered exclusively for unresectable tumors and recurrent meningiomas. It is recommended after subtotal resection (STR) of benign meningioma and for malignant tumors. Several radiation technologies, such as stereotactic radiosurgery (SRS), intensity-modulated radiation therapy (IMRT), fractionated stereotactic radiotherapy (FSRT), and proton-beam therapy (PBT), report promising outcomes for treatment of benign cranial tumors such as pituitary adenomas, craniopharyngiomas, and meningiomas^7,8^. However, no prospective study compares these techniques. In a retrospective study by Han *et al*., comparing SRS, hypofractionated stereotactic radiation treatment (hFSRT), and FSRT, there was no significant difference in terms of clinical and radiological response according to the technique used in the treatment of meningioma of the skull base^9^. However, it is important to adapt the choice of technique to volume and tumor topography. In contrast to conventional fractional radiotherapy, hFSRT is an accurate technique that allows a high dose to be delivered per fraction in a few sessions with a reduction in the margin around the tumor and thus in the surrounding irradiated tissues. It is an ideal technique for treating small lesions with radiobiologically higher doses to maximize local control^10^. The aim of this retrospective and multi-institutional study is to report the experience of hSRT as a curative approach in management of intracranial meningiomas by using different hypofractionated schedules in three regional French radiotherapy departments equipped with a CyberKnife® as exclusive or adjuvant treatment and in local recurrence. We report local control, clinical response, and tolerance.

## Results

The median age at diagnosis of intracranial meningioma was 61 years (range, 21–92). The sex ratio was 2:1 in favor of women, and 97% of patients had a 0–1 performance status (PS). The histological diagnosis was confirmed in 60 cases (48%). Incomplete excision (Simpson grades 3–4) was observed in nine patients (9%) in the primary hFSRT patients group and in seven patients (24%) in the re-irradiated hFSRT group. In this re-irradiated hFSRT group, the median interval between prior irradiation and re-irradiation was 58.6 months (range: 8.8–204.6 months**)**. In the primary hFSRT patients group, the median interval between surgery alone and hFSRT irradiation was 61.4 months (range: 11.2–179 months). The median gross tumor volume (GTV) was 4.84 cm^3^ (0.31–44.70), and the median planning tumor volume (PTV) was 9.35 cm^3^ (0.6–97.3) **(**Table 1**)**. Several dose and fraction schemes have been used, as the most common being 36 Gy/9 fractions in 27% patients, 23.1 Gy/3 fractions in 24% patients, 25 Gy/5 fractions in 17% patients, 21 Gy/3 fractions in 10% patients, and 30 Gy/5 fractions in 10%. The protocol was determined on the basis of tumor volume, proximity to organs at-risk, history of radiation therapy and centre habits. The median clinical and radiologic follow-up duration after completing treatment was 20.2 months (range: 1–77 months).

**Table 1:** Dosimetric results (hypofractionated stereotactic radiation treatment (hFSRT); gross tumor volume (GTV); planning target volume (PTV)).

Dosimetric parameters	Primary hFSRT group (N = 96)	hFSRT Re irradiation (N= 30)
Median Total dose (Gy)	25 (16–40)	25 (12–36)
Median Dose per fraction (Gy)	6 (4–7,7)	5 (4–8)
Median fraction number	5 (3–10)	5 (3–9)
Median GTV/CTV (cm^3^)	4,69 (0,31–42)	5,95 (0,38–44,7)
Median PTV (cm^3^)	9,34 (0,6–97,31)	9,94 (1,44–63,79)
Median Coverage (%)	99 (54–100)	99 (76–100)

### Clinical response

The clinical response evaluation in the overall population was performed in 119 patients. These data were not available for 7 patients. Clinical control of symptom rates was 95% (95% CI: 89–98%) after a median time of 4.4 months (1.1–21.3 months). We noted complete clinical remission symptoms in 37% of patients and improvement in symptoms in 14%. The symptomatology remained stable in 44% of cases. A worsening of the neurological symptoms was observed in 5% of the cases.

### Local control

**Figure 1:**
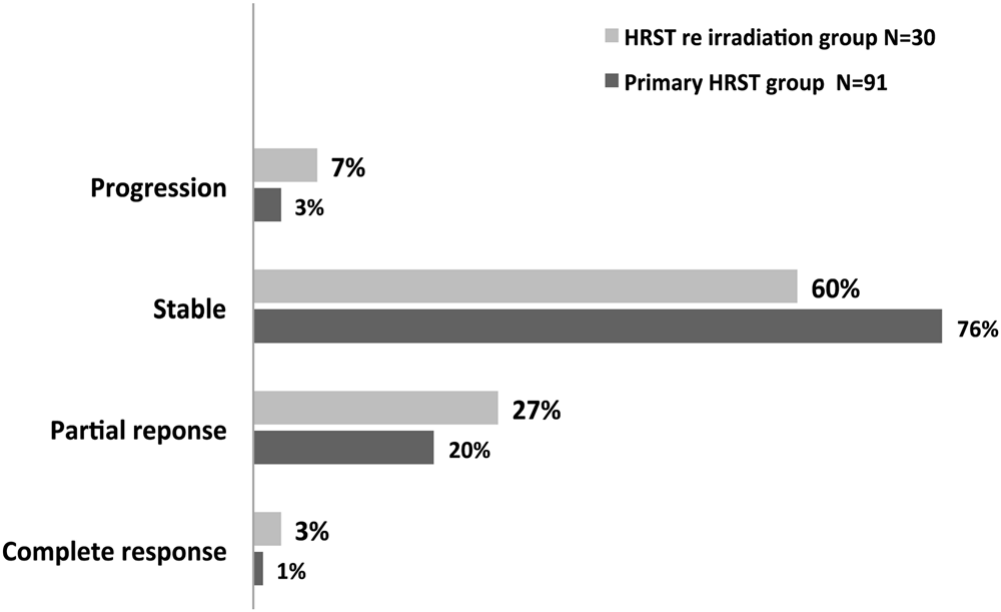
Local control.

**Figure 2:**
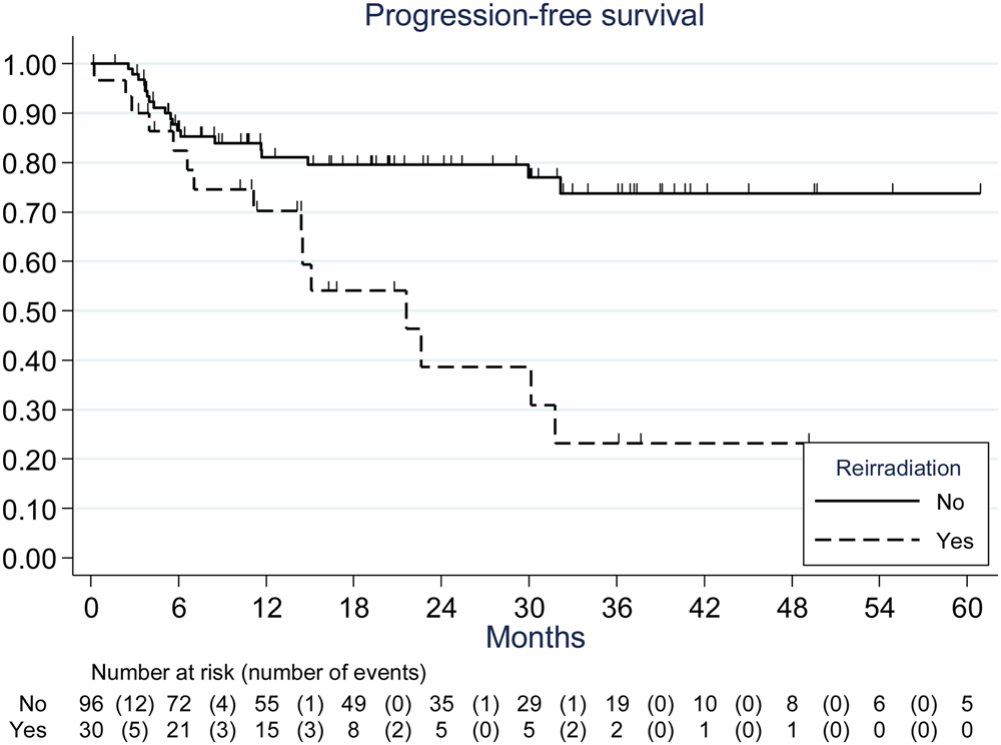
Kaplan-Meier estimates of cumulative progression-free survival (whole population).

The actuarial progression-free survival (PFS) rates in the overall population at 12 and 24 months were 78% (95% CI: 69–85) and 70% (95% CI: 60–79%). After a median follow-up of 20 months, the 24-months local control (LC) rate was 81% (CI 95%: 71–88%) in the primary hFSRT group. In the re-irradiated hFSRT group, the LC rate was 39% (CI 95%: 17–61%). The magnetic resonance imaging (MRI) evaluation best response according to the context of the hFSRT (primary radiotherapy [RT], re-irradiation) is summarized in Fig. 1. Progression-free survival was significantly worse in the case of the re-irradiation hFSRT group (p = 0.002, HR = 3.1, CI 95%: 1.6–6.1) Fig. 2.

**Figure 3:**
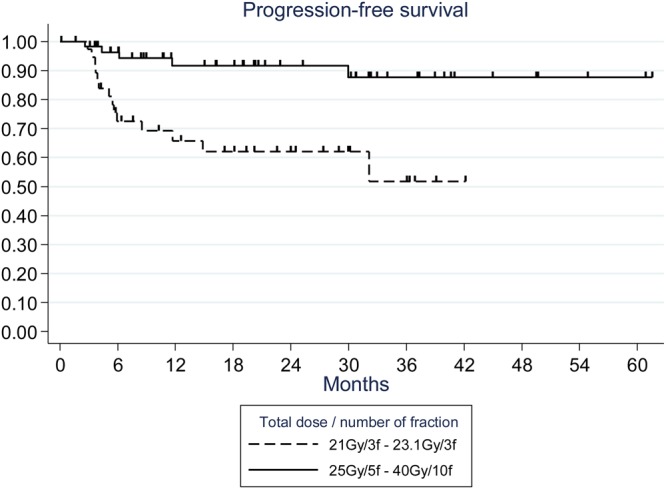
Kaplan-Meier estimates of cumulative progression-free-survival according to different dose/fraction schedules in primary hypofractionated stereotactic radiation treatment group.

On univariate analysis in the primary hFSRT group, the PFS was significantly associated with the radiotherapy schedule; it was significantly lower with the most hypofractionated schedules (21–23 Gy in 3 fractions vs. 25–40 Gy in 5–10 fractions: 24-months PFS = 62% vs. 92%, p = 0.0006) (Fig. 3**)**. A higher total dose (p = 0.014), a lower fraction dose (p = 0.004), and a higher number of fractions (p = 0.008) were significant favorable prognostic factors of PFS. These dosimetric parameters were all defined in the dose scheme variable, thus no multivariate analysis was carried out. In addition, the world health organization (WHO) grade 1 of meningioma appears to be a significant favorable prognostic factor of PFS (p = 0.013). In the re-irradiated hFSRT group, no prognostics factors appeared to be significantly associated with PFS.

### Overall survival

In the total population, the overall survival rate at 24 months was 96% (CI 95%: 88 dosimetric 98%). There was no statistically significant difference between the primary or re-irradiated hFSRT group (p = 0.31).

### Toxicity

#### Early side-effects

The treatment was well tolerated. Patients presented with grade 1 (31%) and/or grade 2 (3%) side-effects, such as asthenia, headaches, temporary focal alopecia, mild erythema, and occasional nausea. We did not observe grade 3, 4, or 5 toxicity. None of the early side-effects required discontinuation of radiotherapy or hospitalization.

#### Late side-effects

**Figure 4:**
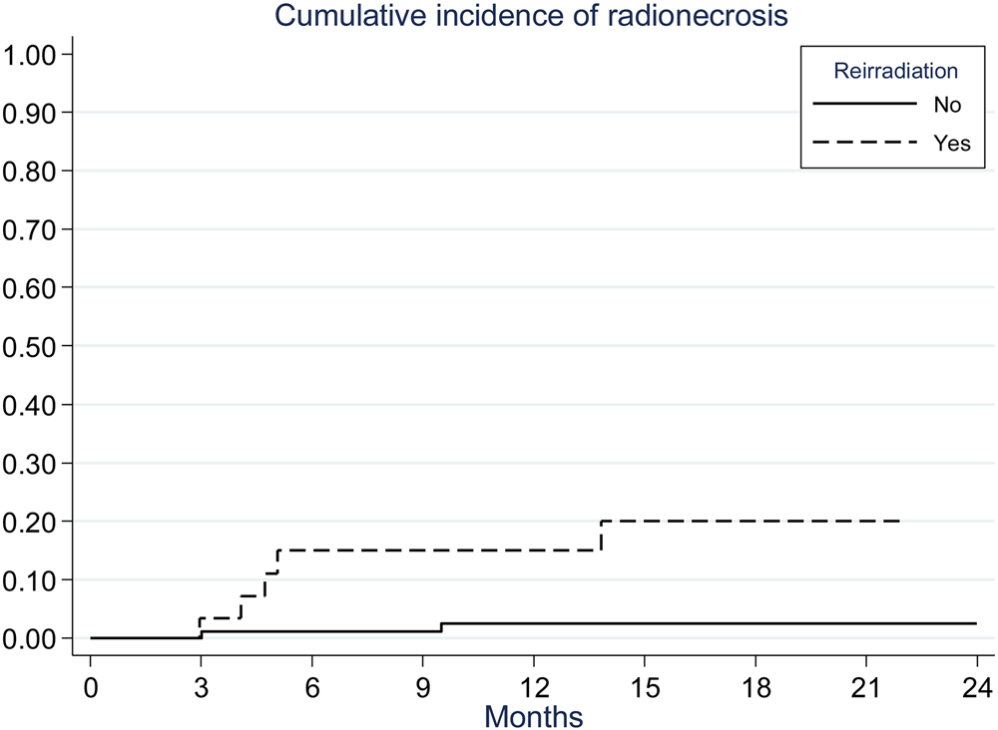
Kaplan-Meier curves of the incidence of radionecrosis.

Two patients presented radionecrosis (RN) in the primary hFSRT group versus five patients in the re-irradiated hFSRT. The diagnosis was exclusively by imaging. The median time to onset of RN for these seven patients was 4.4 months. The incidence of RN was significantly lower in the primary hFSRT group (24-months rate: 2% vs. 20%) than in the re-irradiation hFSRT group (p = 0.002) (Fig. 4). Treatment was symptomatic with corticosteroids in all patients. One patient required an anti-vascular endothelial growth factor treatment. All patients presented a symptom resolution. The low number of RN in the primary and re-irradiated population did not allow a prognosis analysis between the occurrence of RN and dosimetric parameters.

## Discussion

Retrospective studies have demonstrated improved local control with gross total resection over subtotal resection^11^. Moreover, conventional fractionated stereotactic radiotherapy is widely reported as an effective treatment of intracranial meningioma. Single fraction radiosurgery has been described for small tumors (<2.5–3 cm) located distant from critical structures such as the optic nerves. However, it seems this technique cannot be adapted to meningiomas whose volume is greater than 7.5 cc, because abnormally high rates of local recurrence having been reported^12^. Recently hFSRT has become an alternative treatment option for the management of meningiomas, especially of peri-optic lesions within 2 to 3 mm of the optic pathway^8^.

In the primary hFSRT group, 68% patients were symptomatic as a consequence of the tumor location and/or previous surgery in our series. We observed complete or partial clinical response in 51% and stability in 44% cases. Considering the clinical response, stabilization or improvement neurological symptoms has been reported in 69% to 100% of cases after conventional radiotherapy according to series^13,14^. Ho Han and colleagues analyzed the long-term clinical outcome in patients with skull-base meningiomas treated with Gamma Knife SRS and reported a 45% symptomatic improvement in cranial neuropathy^15^. In another study, Han *et al*. compared SRS and FSRT in skull-base meningioma; clinical response was seen in 89%, 100%, and 91% (p = 0.16) in the SRS, hFSRT, and FSRT groups, respectively^9^. In a series of 36 patients treated with Gamma Knife radiosurgery for cavernous sinus meningiomas, Metellus *et al*. reported 58% clinical improvement^16^. In an interesting fact for the re-irradiation hFSRT group, we report a complete and partial clinical response in 14% and 32%, respectively, and stability in 46%, regardless of the location of the meningioma. In our series, 32% of patients treated were asymptomatic. The decision to treat asymptomatic patients was mainly from the detection of documented tumor progression on repeated MRI, which became threatening because of its proximity to critical structures. Indeed, 20% of the meningiomas were located at the base of the skull, of which 13% were located at the cavernous sinus. A recent review, including 22 studies of meningiomas of the cavernous sinus, reported that lesions greater than 2 cm could continue to grow by 10% per year with a risk of becoming symptomatic in 42% of lesions less than 2 cm^17^. In addition, there is no well-established consensus on the management of asymptomatic meningiomas of the skull base, in this case the cavernous sinus meningioma^4,18,19^.

In our series, actuarial local control was 39% (CI 95%: 17–61%) at 20.2 months in the re-irradiation hFSRT group. There are very few data in the literature on stereotactic re-irradiation and this is one of the strengths of our article. Actuarial local control was 81% (CI 95%: 71–88%) in the primary hFSRT group. According to data in the literature about the most commonly used 3 dimensional conformal radiotherapy, the prescription dose was 50–55 Gy in 30–33 fractions with local control rates of 80% and 70% at 10 years and 20 years, respectively; using external beam radiotherapy as adjuvant treatment demonstrated 5-year and 10-year actuarial local control rates of 95% and 80%, respectively^14,20^. The SRS series reported long-term PFS ranging from 93% to 100% for patients treated for skull-base meningiomas^21–23^. In our series the actuarial local control in the primary hFSRT group seems to be lower than reported in the literature. Some patients were treated with more hypofractionated regimens (21–23 Gy in 3 fractions), which are correlated with lower survival without relapse. Further follow-up is needed to confirm these results. Local control (24-months PFS = 92%) with longer regimens (25–40 Gy in 5–10 fractions) is similar to that reported in the literature. Moreover the indications for SRS are mainly limited to small meningiomas located at a distance from the risk structures^19^. A marginal dose of 11–18 Gy has been suggested to treat meningiomas with SRS^22,24^. It is recognized that local recurrence rates are significantly higher for WHO grades II and III meningiomas versus grade I, whereas the role of adjuvant stereotactic radiotherapy (SRT) or SRS in grades II and III meningiomas is not yet well-defined^25^.

In a study of 114 patients, Choi *et al*. reported at five years an LC level of 65% after postoperative stereotactic radiotherapy for grades II and III meningiomas^25^. In a study by Aboukais et al, 27 patients with grade II meningioma, receiving SRS after postoperative radiological progression, showed LC at one, two, and three years of 75%, 52%, and 40%, respectively^26^. The results reported by Pollock *et al*. in 50 patients receiving SRS for grades II and III meningiomas showed LC levels of 85% and 45% at one and five years, respectively^27^. These results suggested that SRS could be an effective therapeutic option in grade II progressive meningiomas. On the other hand, the WHO grade of meningioma appears as a significant prognostic factor of PFS in univariate analysis with better PFS for grade I versus grades II–III in our series.

We were able to compare schedules by using different doses and fractions. We observed that the most hypofractionated schedule (such as 21 Gy/3 fractions and 23.3 Gy/3 fractions) were associated with lower LC at 24 months on the order of 62%. This result was statistically significant compared to other more fractionated regimens (25–40 Gy in 5–10 fractions), in which LC was 92% (p = 0.0006). Despite its short follow-up the originality of this study consists in this finding. To the best of our knowledge no data in the literature has directly compared different schedules of hFSRT. The series of Adler & al. and Colombo & al. reported that during hFSRT of peri-optic meningioma, tumor progression was associated with a loss of vision in patients treated with doses of 21 Gy/3 fractions^28,29^. It is therefore possible that a larger prospective study comparing different dose and fraction regimens may provide a robust result for detecting with statistical power the clinically significant benefit.

Meningioma is a benign pathology in most cases, which means that the life expectancy of patients can be long. The rate of adverse events after complete resections of skull-base meningiomas can reach up to 60%^4,19^. Choosing to treat with hFSRT must be well justified and requires long-term follow-up^30^. In the present study, the follow-up was relatively short and showed low rates of acute toxicity. No patient experienced greater than grade 3 toxicity. Events of late toxicity observed were acceptable in the primary hFSRT group, with radionecrosis incidence of 2% versus 20% in the re-irradiation hFSRT group. This difference was statistically significant. Prognosis analysis of the occurrence of radionecrosis was not performed because of the low number of RN in our population. Rates of acute and late toxicity were generally lower than those reported in several studies after hFSRT, in which the acute toxicity rate climbed from 5% to 48%, with a late toxicity rate of 8% to 35%^31–33^. In a study of SRS for intracranial meningioma, the authors report comparable rates of acute toxicity, with 3.5% to 5% of patients presenting with toxicity^15,23^. In addition, newly developed nerve paralysis seemed to be more frequent with SRS^34^.

Several limitations concerning the assessment of the antitumor efficacy of hFSRT in the present series are possible, such as the retrospective nature of the study and the short follow-up. Our population is heterogeneous; this is a consequence of several factors, such as the diversity of indications of hFSRT in the primary irradiation (adjuvant, exclusive) and re-irradiation contexts and different protocols of prescription of doses and fractionation used in the centers. Prolonged follow-up is needed to obtain robust evaluation of tumor control and late toxicity after hFSRT of intracranial meningiomas. We were unable to perform stratified analyses of grades, hFSRT indication type, and meningioma topography due to the low number of events and low numbers per subgroup. A longer follow-up would be necessary to justify a large adoption of this technique in the management of skull-base meningiomas.

## Materials and Methods

### Patient selection and tumor characteristics

The patients analyzed in this study were pooled from three French institutions of radiotherapy: Lille, Nancy, and Caen. One hundred twenty-six patients were included from December 2008 to June 2016. All patients were treated with CyberKnife^®^ (Accuray Inc., Sunnyvale, CA, USA) for intracranial meningiomas, exclusively with hFSRT, adjuvant hFSRT, or after local relapse. Eighty-six patients (68%) were symptomatic before treatment. The diagnosis was performed by imaging in 52% of cases. The inclusion criteria were all intracranial meningiomas, regardless of WHO classification grade, treated exclusively with robotic hFSRT or after a prior surgical resection. Recurrent meningioma after prior radiotherapy was not restricted. Tumors were located at the falx in n = 20 cases (15%), convexity in n = 42 cases (31%), posterior cerebral fossa in 27 cases (20%), skull base in 27 (20%) cases, optic nerves in three cases (2%), and parasagittal in 17 cases (12%). The median number of lesions irradiated was one (1–4 lesions). The largest median lesion diameter was 20 mm (3–57 mm). According to WHO classification, meningiomas were grade 1 in 42 patients (70%), grade 2 in 15 patients (25%), and anaplastic grade 3 in three patients (5%). In the primary hFSRT patients group of 96 patients, hFSRT was delivered as exclusive treatment in 57 patients (59%), postoperatively in 10 patients (11%), and after local progression after prior surgery alone in 29 patients (30%). In this group, meningiomas were predominantly (88%) grade 1 according to the WHO classification. There were 30 patients in the re-irradiation group. 27 patients were treated for meningioma, including 24 patients treated with combination surgery and 3D conformal external radiotherapy and 3 patients treated with HFSRT. Three patients (n = 3) had a history of conventional external radiotherapy for other intracerebral tumors: 1 medulloblastoma (54Gy/1,8Gy per fraction), 1 craniopharyngioma (54Gy/1,8Gy per fraction) and 1 pituitary adenoma (50 Gy/2Gy per fraction). There were more grade 2 (52%) and grade 3 (14%) cases, according to the WHO. Recurrence diagnoses were based on computed tomography (CT) and MRI criteria and were biopsy-proven when feasible. The patients’ medical records were reviewed for clinical data, treatment details, and outcomes. Follow-up was clinical and radiological (MRI) every 3–6 months. MRI and clinical information were available in almost all patients to the date of analysis. The evaluation of the local response was carried out according to the Response Evaluation Criteria in Solid Tumors (RECIST1.1) criteria. The patient and tumor characteristics are presented in Table 2. The Institutional Committee on Human Research of Centre Oscar Lambret (Lille) has approved this retrospective, multi-institutional, transversal study. Informed consent was obtained for all patients. All research was performed in accordance with relevant regulations.

**Table 2:** Patients and tumor characteristics (world health organization (WHO); hypofractionated stereotactic radiation treatment (hFSRT)).

Patients and tumor characteristics	Number (%)
Gender	
Female	86 (68%)
Male	40 (32%)
Median age (range)	61 (21–92)
Median PS (range)	1 (0–2)
Treatment Center	
Center 1	65 (51%)
Center 2	42 (43%)
Center 3	19 (15%)
Diagnosis	
Imaging	66 (52%)
Histopathologically	60 (48%)
WHO Meningioma grade	
I	39(69%)
II	15(26%)
III	3(5%)
Median Tumor volume cm^3^ (range)	4,84 (0,3–44,7)
Median maximal tumor diameter mm (range)	20 (3–57)
Presenting symptoms	
Headache	34 (27%)
Vertigo	9 (7%)
Cranial nerve deficit	10 (8%)
Visual impairment	18 (14%)
Ataxia	7 (6%)
Focal seizures	16 (13%)
Phasic desord**e**r	4 (3%)
Paresis	10 (8%)
Paresthesia	12 (9%)
Decreased hearing	7 (6%)
Location	
Skull base	27 (20%)
Convexity	42 (31%)
Parasagittal	17 (12%)
Falx	20 (15%)
Posterior cerebral fossa	27 (20%)
Optic nerves	3 (2%)
Prior Surgery	64 (51%)
Median interval from prior surgery to hFSRT (months)	61.4 months (11,2–179)
Simpson grade	
I	12 (21%)
II	30 (52%)
III	9 (16%)
IV	7 (12%)
Prior Radiotherapy modality	
External beam radiotherapy	27 (21%)
Robotic hFSRT	3 (3%)
Median interval from prior radiotherapy to re irradiation (months)	58.6 (8.8–204.6)

### hFSRT planning and target volumes

Target delineation was made using a planning CT that was registered with MRI. The GTV or the clinical target volume (CTV) of the tumor’s bed was delineated on computed tomography and/or MRI scans. The GTV or CTV was corrected manually to account for the natural anatomic barriers and preoperative volume. Adding margins of 1–2 mm around the GTVs or CTVs created the PTV.

### Dose protocols

Several hypofractionated schedules of dose and fraction were used according to the practices of the radiotherapy departments. The median dose was 25 Gy (range 12–40 Gy) with median dose per fraction of 5 Gy (range 4–8 Gy). A total dose was prescribed to the 80% isodose line, which covers 95% of PTV. The dose constraints for the organs at risk (OARs) are listed in Table 3. Treatment was delivered every other day.

**Table 3:** Dose constraints for organs at risk in the three centers (fraction (fr)).

Organ-at-risk	Centers	1 fr	3 fr	5 fr	6 fr
Brain	Center 1	Dmax 18 Gy V10<1 cm^3^	Dmax 23 Gy V10<1 cm^3^	—	—
	Center 2	—	—	—	—
	Center 3	Dmax 15Gy V12< 5–7 cm^3^	—	—	—
Brain steam	Center 1	Dmax 12Gy	V18<1 cm^3^	Dmax 22 Gy	—
	Center 2	Dmax 12Gy	Dmax 17Gy	—	—
	Center 3	Dmax 15Gy V10<0,5 cm^3^	Dmax 23Gy V18<0,5 cm^3^	Dmax 31Gy V23< 0,5 cm^3^	—
Optic Nerve	Center 1	V8<0,2 cm^3^	Dmax 14 Gy	V25< 55%	
	Center 2	V8<0,2 cm^3^	V21<0,2 cm^3^ V27<0,003 cm^3^	V20<0,2 cm^3^ V25<0,003 cm^3^	V10<0.5 cm^3^ V15<0.2 cm^3^
	Center 3	V8<0,2 cm^3^ Dmax 10Gy	V15<0,2 cm^3^ Dmax = 17Gy	V20<0,2 cm^3^ Dmax 25Gy	V21,5<0,2 cm^3^ Dmax 27Gy
Optic Chiasma	Center 1	Dmax 10 Gy V8< 0,2 cm^3^	Dmax 10,5 Gy V15< 0,2 cm^3^	Dmax 25 Gy V20< 0,2 cm^3^	—
	Center 2	V8<0,2 cm^3^	V21<0,2 cm^3^ V27<0,003 cm^3^	V20<0,2 cm^3^ V25<0,003 cm^3^	V10<0.5 cm^3^ V15<0.2 cm^3^
	Center 3	V8<0,2 cm^3^ Dmax = 10Gy	V15<0,2 cm^3^ Dmax = 17Gy	V20<0,2 cm^3^ Dmax = 25Gy	V21,5<0,2 cm^3^ Dmax = 27Gy
Cochlea	Center 1	—	Dmax20Gy	—	—
	Center 2	Dmax 12 Gy	Dmax 30 Gy	Dmax 27Gy	Dmax 20 Gy
	Center 3	Dmax 12 Gy	Dmax 20 Gy	Dmax 27Gy	Dmax 30 Gy
Lens	Center 1	—	—	Dmax 6 Gy	—
	Center 2	—	Dmax 6 Gy	Dmax 6 Gy	Dmax 6 Gy
	Center 3	Dmax 3 Gy	Dmax 5 Gy	Dmax 7 Gy	Dmax 7 Gy

### Follow-up evaluation

After treatment, the patient follow-up visits, including physical examination and cranial MRI, were held every three months for the first year, every six months for the next year, and annually thereafter.

### Endpoints and statistical analysis

Statistical analyses were performed with Stata 13.1 (StataCorp, 2013; Stata Statistical Software, release 13; StataCorp LP, College Station, TX). Survival endpoints were estimated from the last day of hFSRT, using the Kaplan-Meier method and compared between groups using log-rank test. Local control rate (LC) was defined as time interval until local recurrence diagnosed by MRI; patients free from local recurrence were censored at the date of last follow-up examination or death. PFS was defined as time interval until progression or death; patients alive without progression were censored at the date of the last follow-up examination. Overall survival (OS) was defined as the time interval until death from any cause, patients alive were censored at the date of the last follow-up examination. A prognosis analysis was performed on PFS in the primary HSRT group and in the re-irradiation group separately. Univariate and multivariate analysis were realized using the log-rank test and the proportional hazards Cox model. A *p* value <0.05 was chosen as the significance threshold. Early (<90 days after radiotherapy completion) and late side-effects (>90 days) were graded according to the Common Terminology Criteria for Adverse Events, version 4.0, grading system. Cumulative incidence of radionecrosis was estimated using the Kaplan-Meier method as the time interval from the last day of hFSRT to the occurrence of radionecrosis.

## Conclusion

We present one of the largest series of patients treated with hypofractionated stereotactic radiotherapy to date. Radiotherapy regimen appears to be crucial. The indication of hFSRT can be considered a promising therapeutic option in primary meningioma treatment with special attention to sufficient follow-up. Obviously, prospective future studies with longer follow-up and more patients are warranted to confirm our findings.

## Data Availability

The datasets generated during and/or analysed during the current study are available from the corresponding author on reasonable request.
